# Association Between the Frailty and New-Onset Atrial Fibrillation/Flutter Among Elderly Hypertensive Patients

**DOI:** 10.3389/fcvm.2022.881946

**Published:** 2022-05-06

**Authors:** Fei Hang, Jieruo Chen, Zefeng Wang, Jiafu Yan, Yongquan Wu

**Affiliations:** Department of Cardiology, Beijing Anzhen Hospital, Capital Medical University, Beijing, China

**Keywords:** frailty, atrial fibrillation/flutter (AF), systolic blood pressure intervention trial (SPRINT), hypertension, elderly patients

## Abstract

**Background:**

Frailty was found to be common in patients with atrial fibrillation/flutter (AF), but there was still a lack of evidence regarding the relationship between frailty and new-onset AF.

**Methods:**

We performed a *post hoc* analysis of data from the Systolic Blood Pressure Intervention Trial (SPRINT). In addition, we evaluated the relationship between baseline frailty status and new-onset AF in older adult patients with hypertension.

**Results:**

In total, 7,316 participants were included in our analysis, and a total of 115 new-onset AF occurred during an average of 3.54 years of follow-up. Using SPRINT frailty index criteria, 1,535 fit, 4,041 less fit, and 1,740 frailty were enrolled. Compared with other groups, the incidence of new-onset AF in the frailty group was significantly higher. We constructed three Cox models to assess the relationship between the frailty status (fit group as reference) and new-onset AF. Participants with frailty had a significantly higher risk of new-onset AF compared with the fit group in all the models we used. We combined the fit group and the less fit group into a no frailty group to assess the impact of frailty on new-onset AF in various subgroups. After full adjustment (Model 3), frailty remained associated with the increased risk of new-onset AF compared with the no frailty group [hazard ratio [*HR*] = 2.09, *95% CI*:(1.41, 3.09), *p* < 0.001]. Additionally, we examined the frailty index as continuous variable to assess the relationship between the frailty index and new-onset AF. The smooth curve showed that log HR appeared to increase linearly. And there was a significant interaction between baseline systolic blood pressure (SBP) categories and frailty on the risk of new-onset AF (*p for interaction* = 0.030).

**Conclusion:**

This study found baseline frailty status was a strong independent risk factor for new-onset AF among older adult patients with hypertension. Screening for frailty should be considered in older adult patients with hypertension to prevent new-onset AF.

## Introduction

Atrial fibrillation/flutter (AF) is the most common cardiac arrhythmia and is prevalent in older adults, with a 31% increase in global incidence over the past 2 decades (1). The patient with AF was at higher risk of developing stroke and heart failure, which can lead to a deterioration in their functional status and eventually to a state of frailty (2). Frailty is a common geriatric syndrome that reflects a state of vulnerability to adverse health events, marked by abnormal responses to stressors and decreased ability to maintain homeostasis (3, 4). Frailty was found to be common in patients with AF and may contribute to the pathophysiological development of cardiovascular diseases (5).

Several previous studies found a significantly higher prevalence of frailty in patients with AF compared with those without AF (5–7). However, other studies found no statistically significant association between frailty and prevalent AF (6). Moreover, there was still a lack of data regarding the relationship between frailty and new-onset AF, which may provide important guidance for AF prevention in the older adult with hypertension. Therefore, this study aims to examine whether baseline frailty was associated with the development of AF among older adult patients with hypertension. Understanding the association between frailty status and new-onset AF among older adult patients with hypertension may present important guidance for AF prevention in clinical settings.

## Materials and Methods

Data used in this analysis were derived from the SPRINT dataset, available at the National Heart, Lung, and Blood Institute BioLINCC data repository. Researchers could visit https://biolincc.nhlbi.nih.gov/home/to apply for access to the public database.

### Study Population and Design

The present analysis was based on the SPRINT cohort. SPRINT was a randomized, controlled, open-label trial conducted at 102 clinical sites in the United States and approved by all institutional review boards at the respective trial sites. The rationale, design, and main result of the SPRINT have been previously published (8, 9). Briefly, the SPRINT cohort began in November 2010 with the enrollment of 9,361 participants with hypertension at high cardiovascular risk. All participants provided informed consent to participate in this trial.

**FIGURE 1:**
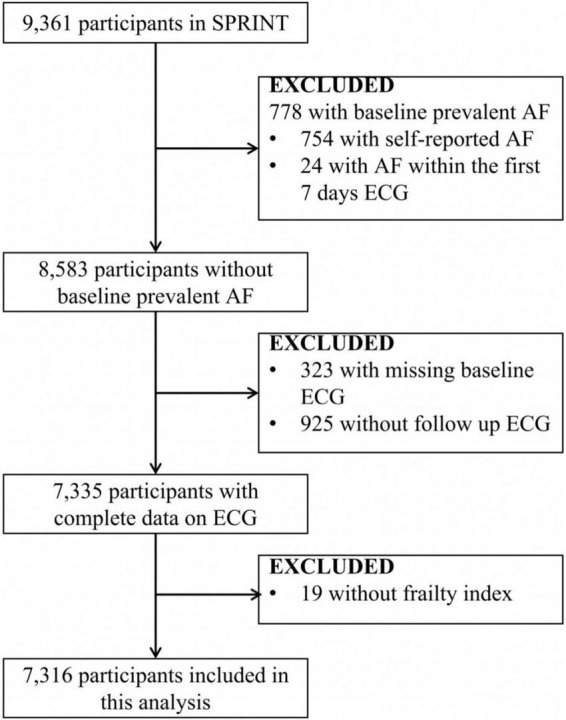
Inclusions and exclusions of the participants included in the analysis. AF indicates atrial fibrillation/flutter. SPRINT, Systolic Blood Pressure Intervention Trial.

Our analysis aimed to assess the relationship between baseline frailty status and new-onset AF in SPRINT participants. Participants with preexisting AF, missing baseline and follow electrocardiogram (ECG), and no frailty index were excluded from the analysis. The detailed inclusion/exclusion process is presented below (Figure 1). Finally, 7,316 participants were included in the analysis.

### Electrocardiogram Ascertainment

A GE MAC 1200 electrocardiograph (GE, Milwaukee, WI, United States) with 10 mm/mv calibration and a speed of 25 mm/s was used to collect digital ECG data. ECG examinations were performed at baseline, year 2 follow, year 4 follow, and closeout follow (8). All ECG readings were adjudicated by the Epidemiological Cardiology Research Center at Wake Forest School of Medicine (Winston-Salem, NC, United States) (8, 10). Digital ECG data were collected in the SPRINT dataset.

### Baseline Frailty Status Assessment

Baseline frailty status was assessed by the SPRINT 36-item (37-item among the subset of participants 75 years of age and older) frailty index criterion (11). SPRINT frailty index criteria included blood pressure measurements, self-report comorbidities, laboratory examinations, self-assessments of depression from the 9-item Patient Health Questionnaire (PHQ-9), self-assessment of health from the Veterans RAND 12-Item Health Survey (VR-12), global cognitive status based on Montreal Cognitive Assessment (MoCA), and information from two other cognitive screening instruments (11). Each item in SPRINT frailty index criteria was weighted equally. The frailty index was the mean of the scores of all non-missing items, and participants without at least 30 non-missing items were excluded from this analysis. The baseline frailty status was classified as fit (frailty index ≤0.10), less fit (0.10< frailty index ≤0.21), and frailty (frailty index >0.21) (11).

### Study Outcome

The outcome of this analysis was the first occurrence of new-onset AF on ECG in SPRINT participants without preexisting AF. The participants with self-report AF at baseline (754 patients) or detection of AF within the first 7 days of ECG (24 patients) were defined as having preexisting AF. Considering the outcome of this analysis was new-onset AF, participants without baseline (323 patients), or follow-up (925 patients) ECG were excluded from this analysis.

### Statistical Analysis

The baseline characteristics of participants were compared by baseline frailty status. Descriptive statistics were calculated using mean ± standard deviation (SD) or median (P25, P75) for continuous variables and frequency count (percentage) for categorical variables. An ANOVA or non-parametric test was used for inter-group differences of continuous variables, and the chi-square test or Fisher’s test was used for categorical variables. Three Cox proportional hazards models were used to estimate the hazards ratio (*HR*) and calculate a 95% confidence interval (*95% CI*), quantifying the relationship between baseline frailty status (fit group as reference) and new-onset AF. The variables with baseline difference and variables that might affect the outcome were included as covariates. Model 1 was unadjusted; Model 2 was adjusted for age, sex, race, and body mass index; and Model 3 was fully adjusted for age, sex, race, body mass index, treatment arms, baseline systolic blood pressure (SBP), heart rate, serum creatinine, urine albumin/creatinine ratio, estimated glomerular filtration rate (GFR), total cholesterol, triglycerides, high-density lipoprotein- cholesterol (HDL-C), glucose, smoking status, statin use, aspirin use, previous cardiovascular disease (CVD), previous chronic kidney disease (CKD), self-reported diabetes, self-reported stroke or transient ischemic attacks (TIA), Sokolow-Lyon Index (SV1 amplitude + RV5/V6 amplitude), anemia, and Framingham 10-year CVD risk. We used the graphical methods *via* the scaled Schoenfeld residuals to examine the proportional hazard assumption. Kaplan–Meier analyses were performed for the cumulative incidence of new-onset AF by baseline frailty status after adjustment for all covariates in Model 3. The dose-response relationship between frailty index and new-onset AF was conducted using the generalized additive model and fitting smooth curve (restricted cubic splines). The relationship between frailty status (frailty vs. no frailty) and outcome according to various subgroups was assessed with stratified analysis and an interaction test. All analyses were performed using the statistical software package R (The R Foundation).^1^ Statistical significance was set at *p* < 0.05.

## Results

To assess the association between frailty status and new-onset AF, 7,316 participants (35.9% women; mean age: 67.56 ± 9.21 years) were included in this analysis. A total of 115 new-onset AF events occurred during an average of 3.54 years of follow-up. The details of the inclusion and exclusion of the study participants are shown in Figure 1.

The characteristics of baseline participants according to baseline frailty status are shown in Table 1. Using SPRINT frailty index criteria, 1,535 fit, 4,041 less fit, and 1,740 frails were enrolled. The frailty group was older, had higher BMI, SBP, heart rate, serum creatinine, urine albumin-creatinine ratio (uACR), and HDL cholesterol levels, were more likely to be smokers, were more likely to be black, were more likely to use statins and aspirin, had higher rates of CVD and CKD, had higher rates of self-reported diabetes, stroke, or TIA, and anemia compared with the fit group and less fit group.

**TABLE 1:** Baseline characteristics and crude outcome of the Systolic Blood Pressure Intervention Trial (SPRINT) participants by baseline frailty status.

Variables	Frailty status			*P-*Value
	Fit	Less fit	Frailty	
	FI ≤ 0.10	0.10 < FI ≤ 0.21	FI > 0.21	
Number of participants	1535	4041	1740	–
Frailty Index	0.07 ± 0.02	0.15 ± 0.03	0.27 ± 0.05	<0.001
Treatment				
Intensive, n (%)	764 (49.77%)	1997 (49.42%)	880 (50.57%)	0.772
BMI(Kg/m2), mean ± SD	28.47 ± 4.59	29.92 ± 5.69	31.04 ± 6.46	<0.001
Age, y				
Overall	66.05 ± 8.07	67.79 ± 9.09	68.21 ± 10.24	<0.001
≥75 y, n (%)	269 (17.52%)	1100 (27.22%)	565 (32.47%)	<0.001
Female, n (%)	420 (27.36%)	1442 (35.68%)	767 (44.08%)	<0.001
Race, n (%)				<0.001
Non-hispanic white	920 (59.93%)	2373 (58.72%)	850 (48.85%)	
Non-hispanic black	403 (26.25%)	1159 (28.68%)	692 (39.77%)	
Hispanic	178 (11.60%)	439 (10.86%)	175 (10.06%)	
Other	34 (2.21%)	70 (1.73%)	23 (1.32%)	
Baseline blood pressure, mm Hg				
Systolic, mean ± SD	137.46 ± 13.43	139.68 ± 15.38	141.30 ± 16.78	<0.001
Diastolic, mean ± SD	78.02 ± 9.39	78.30 ± 11.86	78.82 ± 13.39	0.138
Heart rate, bpm, mean ± SD	65.06 ± 10.53	66.09 ± 11.40	67.33 ± 12.38	<0.001
SBP categories, n (%)				<0.001
≤132 mmHg	576 (37.52%)	1328 (32.86%)	534 (30.69%)	
>132 mmHg to <145 mmHg	529 (34.46%)	1340 (33.16%)	546 (31.38%)	
≥ 145 mmHg	430 (28.01%)	1373 (33.98%)	660 (37.93%)	
Serum creatinine, mg/dL, mean ± SD	0.97 ± 0.19	1.05 ± 0.31	1.19 ± 0.43	<0.001
Urine albumin/creatinine ratio, mg/g Cr, median (Q1-Q3)	7.27 (4.90–12.62)	9.09 (5.65–19.57)	13.00 (6.49–39.70)	<0.001
Estimated GFR, mL min–1 1.73 m–2, median (Q1-Q3)	76.49 (68.17–86.91)	71.81 (59.04–85.13)	63.07 (48.24–81.88)	<0.001
Fasting total cholesterol, mg/dL, mean ± SD	192.16 ± 34.08	191.62 ± 41.38	189.09 ± 45.95	0.055
Fasting total triglycerides, mg/dL, median (Q1-Q3)	105.00 (78.00–147.00)	107.00 (77.00–150.00)	110.00 (78.00–158.00)	0.123
Fasting HDL cholesterol, mg/dL, median (Q1-Q3)	49.00 (43.00–56.00)	49.00 (43.00–56.00)	51.00 (42.00–62.00)	<0.001
Fasting glucose, mg/dL, mean ± SD	98.30 ± 12.14	99.03 ± 13.12	98.73 ± 15.00	0.184
Statin use, n (%)	544 (35.58%)	1716 (42.73%)	853 (49.31%)	<0.001
Aspirin use, n (%)	735 (48.04%)	1991 (49.36%)	943 (54.29%)	<0.001
Smoking status, n (%)				<0.001
Never smoked	920 (59.93%)	1754 (43.41%)	613 (35.23%)	
Former smoker	531 (34.59%)	1778 (44.00%)	731 (42.01%)	
Current smoker	81 (5.28%)	506 (12.52%)	394 (22.64%)	
Previous CVD, n (%)	115 (7.49%)	694 (17.17%)	531 (30.52%)	<0.001
Previous CKD, n (%)	100 (6.51%)	1075 (26.60%)	788 (45.29%)	<0.001
Self-reported diabetes, n (%)	6 (0.39%)	60 (1.48%)	50 (2.87%)	<0.001
Self-reported stroke or TIA, n (%)	32 (2.08%)	112 (2.77%)	65 (3.74%)	0.016
Anemia, n (%)	110 (7.17%)	430 (10.64%)	321 (18.45%)	<0.001
Sokolow-Lyon Index	21.35 ± 8.29	20.92 ± 8.52	20.81 ± 9.16	0.154
Framingham 10-y CVD risk score,%, median (Q1-Q3)	17.38 (11.94–24.19)	17.67 (11.98–25.46)	17.14 (11.64–26.62)	0.161
New-Onset AF, n (%)	13 (0.85%)	53 (1.31%)	49 (2.82%)	<0.001

*FI, frailty index; BMI, body mass index; SD, standard deviation; CVD, cardiovascular diseases; CKD, chronic kidney disease; TIA, Transient Ischemic Attacks; AF, atrial fibrillation/flutter.*

### The Association Between Frailty Status and New-Onset Atrial Fibrillation/Flutter

**FIGURE 2:**
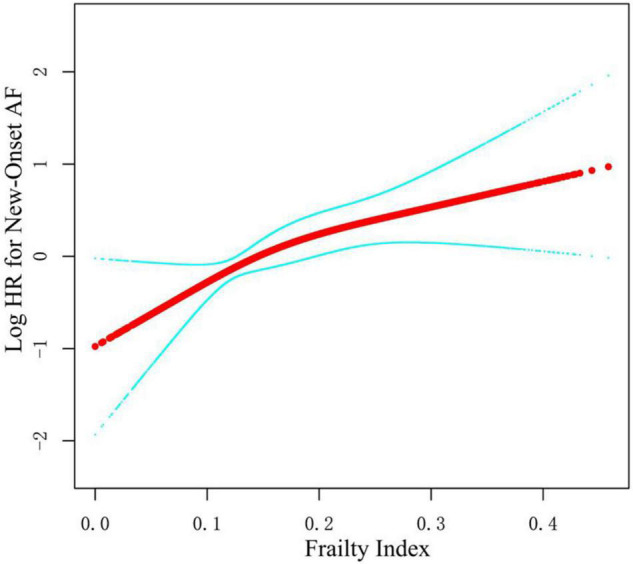
Smooth spline curves of frailty index for the logarithm of hazard ratio of new-onset AF. Red line represents references for hazard ratios (*HR*s), and blue lines represent 95% confidence interval (*CI*). Adjusted for all covariates in the Model 3.

As shown in Table 2, we constructed three Cox models to assess the relationship between the frailty status (using the fit group as a reference) and new-onset AF. There was no statistically significant difference in the risk of new-onset AF between the less fit group vs. the fit group in three models [Model 1: *HR* = 1.40, *95% CI*:(0.82, 2.39), *p* = 0.221; Model 2: *HR* = 1.26, *95% CI*: (0.73, 2.16), *p* = 0.404; and Model 3: *HR* = 1.14, *95% CI*:(0.63, 2.07), *p* = 0.658]. Participants with frailty had a significantly higher risk of new-onset AF compared with the fit group in unadjusted [Model 1: *HR* = 3.16, *95% CI*:(1.83, 5.44), *p* < 0.001] and slightly adjusted model [Model 2: *HR* = 2.87, *95% CI*:(1.65, 4.99), *p* < 0.001]. After full adjustment, frailty still had a statistically significant association with the increased risk for new-onset AF [Model 3: *HR* = 2.21, *95% CI*: (1.15, 4.27), *p* = 0.018]. In addition, we examined the frailty index as continuous variable to assess the relationship between frailty index and new-onset AF. The fitting smooth curve in Figure 2 showed that the log HR appeared to increase linearly.

**TABLE 2:** Association between frailty status and new-onset AF in unadjusted and adjusted models.

	Hazard ratio (*95%CI*) *P*-Value		

**Frailty status**	**Model 1**	**Model 2**	**Model 2**
**Fit**	**Ref.**	**Ref.**	**Ref.**
Less fit	1.40 (0.82, 2.39) *P* = 0.221	1.26 (0.73, 2.16) *P* = 0.404	1.14 (0.63, 2.07) *P* = 0.658
Frailty	3.16 (1.83, 5.44) *P* < 0.001	2.87 (1.65, 4.99) *P* < 0.001	2.21 (1.15, 4.27) *P* = 0.018

*Model 1: adjusted for none. Model 2: adjusted for age, sex, race, and BMI. Model 3: adjusted for age, sex, race, BMI, treatment arms, baseline systolic blood pressure, heart rate, serum creatinine, urine albumin/creatinine ratio, estimated glomerular filtration rate (GFR), total cholesterol, triglycerides, high-density lipoprotein cholesterol (HDL-C), glucose, smoking status, statin use, aspirin use, previous CVD, previous CKD, self-reported diabetes, self-reported stroke or TIA, Sokolow-Lyon Index, anemia and Framingham 10-year CVD risk.*

### Subgroup Analyses of the Impact of Frailty on New-Onset Atrial Fibrillation/Flutter

**FIGURE 3:**
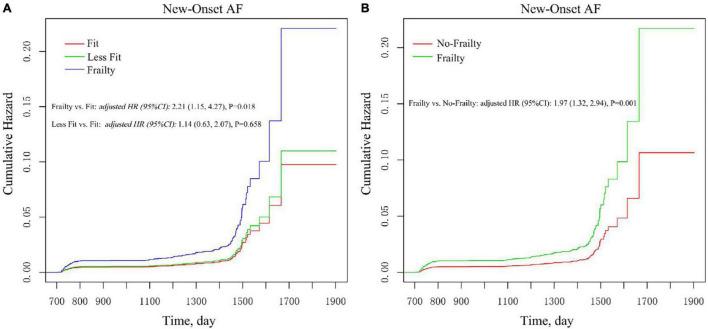
Multivariable-adjusted Kaplan–Meier estimation of new-onset AF by baseline frailty status. **(A)** Frailty vs. less fit vs. fit. **(B)** Frailty vs. no frailty. Adjusted for all covariates in the Model 3.

As shown in Figure 3A, there was no statistically significant difference between the fit group and the less fit group in the cumulative estimation of new-onset AF (Model 3 was used). Therefore, we combined the fit group and the less fit group into a no frailty group to assess the impact of frailty on new-onset AF in various subgroups. After full adjustment (Figure 3B and Table 3), frailty remained associated with the increased risk of new-onset AF compared with the no frailty group [*HR* = 1.97, *95% CI*: (1.32, 2.94), *p* = 0.001]. As shown in Table 3, the impact of frailty on new-onset AF was consistent in prescribed subgroups: sex (male vs. female), age (<75 vs. ≥ 75 years), previous CVD (yes vs. no), previous CKD (yes vs. no), Framingham 10-year CVD risk (<15 vs. ≥ 15%), aspirin use (yes vs. no), and statin use (yes vs. no). All *p-*values for interaction were >0.05. However, there was a significant interaction between baseline SBP categories and frailty on the risk of new-onset AF (*P for interaction* = 0.030). Frailty was associated with increased risk of new-onset AF in individuals with SBP ≥ 145 mmHg [*HR* = 3.12, *95% CI*: (1.69, 5.76), *p* < 0.001] and with the level of 132–145 mmHg [*HR* = 2.97, *95% CI*:(1.50, 5.89), *p* = 0.002], while the relationship between frailty and new-onset AF was not significant among those with SBP ≤132 mmHg [*HR* = 0.97, *95% CI*: (0.46, 2.06), *p* = 0.932].

**TABLE 3:** Subgroup analysis of the risk of new-onset AF between frailty and no-frailty.

Subgroup	Frailty		No-frailty		*HR* (*95%CI*)	*P*-Value	*P* for Interaction
	Event, n/N	Event rate (95%CI)/1000 PY	Event, n/N	Event rate (95%CI)/1000 PY			
Overall	56/1740	9 (2, 377)	79/5576	4 (1, 159)	1.97 (1.32, 2.94)	0.001	–
**Sex**							0.631
Male	37/937	12 (2, 467)	58/3714	4 (1, 176)	1.89 (1.19, 3.03)	0.008	
Female	19/767	7 (1, 299)	21/1862	3 (1, 130)	2.30 (1.17, 4.54)	0.016	
**Age group**							0.946
<75	23/1175	6 (1, 231)	44/4207	3 (0, 117)	2.03 (1.17, 3.52)	0.012	
≥ 75	33/565	18 (3, 714)	35/1369	7 (1, 302)	2.08 (1.22, 3.56)	0.008	
**SBP categories**							0.030
≤132	12/534	7 (1, 281)	32/1904	5 (1, 191)	0.97 (0.46, 2.06)	0.932	
>132 to <145	19/546	10 (2, 421)	23/1869	3 (1, 141)	2.97 (1.50, 5.89)	0.002	
≥145	25/660	11 (2, 451)	24/1803	4 (1, 155)	3.12 (1.69, 5.76)	<0.001	
**Treatment arms**							0.528
Standard	31/860	11 (2, 429)	44/2815	4 (1, 177)	2.21 (1.34, 3.63)	0.002	
Intensive	25/880	8 (1, 339)	35/2761	4 (1, 144)	1.75 (0.97, 3.15)	0.063	
**Previous CVD**							0.793
Yes	24/531	13 (2, 548)	18/809	6 (1, 265)	1.94 (1.20, 3.14)	0.007	
No	32/1209	8 (1, 312)	61/4767	4 (1, 143)	2.15 (1.12, 4.13)	0.021	
**Previous CKD**							0.083
Yes	26/788	10 (2, 395)	24/1175	6 (1, 236)	1.34 (0.73, 2.48)	0.348	
No	30/952	9 (2, 374)	55/4401	3 (1, 141)	2.64 (1.63, 4.30)	<0.001	
**CVD risk**							0.809
<15%	13/708	5 (1, 225)	20/2168	3 (0, 105)	1.90 (0.85, 4.21)	0.116	
≥15%	43/1032	12 (2, 493)	59/3408	5 (1, 197)	2.11 (1.35, 3.32)	0.001	
**Aspirin use**							0.669
Yes	36/943	11 (2, 451)	43/2726	4 (1, 178)	2.14 (1.29, 3.54)	0.003	
No	20/794	7 (1, 304)	36/2838	4 (1, 144)	1.80 (0.97, 3.34)	0.061	
**Statin use**							0.830
Yes	28/853	4 (1, 144)	37/2260	5 (1, 186)	1.93 (1.13, 3.29)	0.015	
No	26/877	9 (1, 353)	41/3285	3 (1, 141)	2.09 (1.21, 3.60)	0.008	

*PY, person year; CVD RISK, ramingham 10-y CVD risk. All covariates in the model 3 except stratification itself were adjusted.*

## Discussion

This study found a statistically significant association between baseline frailty status and new-onset AF in older adult patients with hypertension. A significantly higher risk of new-onset AF was found in hypertensive older adults with frailty compared with those who were fit. The impact of frailty on new-onset AF was consistent in different sub-groups. The recognition of frailty status can help cardiologists prevent the development of AF and establish a better prognosis in older adult patients with hypertension. Screening for frailty should be considered in older patients with hypertension to prevent new-onset AF.

Frailty has been reported to be bi-directionally associated with many cardiovascular diseases (6). However, the association between frailty and AF was still under debate. A systemic review identified 10 studies examining the relationship between AF and frailty, defined in various ways, and suggested a high prevalence of frailty among patients with AF (1). However, 9 out of the 10 included studies were cross-sectional studies, which provide little information on the direction of such a relationship, and the only prospective study was not specifically designed to examine the effect of frailty on new-onset AF, with only data on baseline frailty prevalence among patients with AF available. Moreover, results from previous studies were inconclusive. A prospective study on the older adult cohort found a higher prevalence of frailty among patients with AF (12). However, data from the Cardiovascular Health Study reported no statistically significant association between prevalent AF and frailty [odds ratio [*OR*] = 1.90, *95% CI*: (0.82, 4.39), *p* = 0.325] (5).

Only one previous study by Orkaby et al. using data from the Framingham Heart Study Offspring Cohort, examined the relationship between frailty and new-onset atrial fibrillation. However, the *HR* of incident AF due to frailty was 1.22 (95% *CI*, 0.95–1.55), and the association between frailty and incident AF was not statistically significant (6). The difference in results between our study and theirs might be attributable to different criteria for assessing frailty. The variables in Fried frailty criteria (13), which were applied in the Framingham Heart Study Offspring Cohort, included low physical activity level, low gait speed, self-report of exhaustion, weight loss, and weakness (grip strength). However, the SPRINT frailty criteria included 37 items, including not only variables similar to all the Fried frailty criteria but also laboratory tests, blood pressure measurements, assessments of cognitive function, and daily living and mental health self-assessments to assess frailty more objectively and comprehensively. In our study, we found baseline frailty in older adults with hypertension had a statistically significant association with the increased risk for new-onset AF. Of note, this was the first study reporting frailty as an independent risk factor in developing AF. In addition, our study assessed the effect of baseline frailty on new-onset AF, whereas only new-onset atrial fibrillation was included in the study by Orkaby et al. We also evaluated frailty status based on Fried frailty criteria among participants 75 years and older. There was no statistical difference in the incidence of new-onset AF across different frailty statuses (Supplementary Table 1). Consistent with the study of Orkaby et al., patients with frailty assessed by Fried frailty criteria had a higher but non-significant risk of new-onset AF [*HR (95% CI)*: 2.05 (0.83, 5.06)] (Supplementary Table 2).

Our analysis also found an interaction between baseline SBP categories and frailty on the risk of new-onset AF. Frailty was not associated with the occurrence of AF in individuals with SBP ≤132 mmHg. However, frailty was associated with the increased risk of new-onset AF among participants with baseline SBP >132 mmHg, and the *HR* of the new-onset AF due to frailty was higher in the individuals with SBP ≥ 145 mmHg, as compared with participants with an SBP level of 132–145 mmHg. Chronic increased blood pressure leads to the increased hemodynamic burden of the left atrium, and the resultant complex structural, architectural, contractile, or electrophysiological changes of the left atrium could trigger the occurrence of atrial fibrillation (14, 15). Previous population-based observational studies have reported a linear trend between the baseline SBP levels and the occurrence of AF. A cardiovascular survey of healthy Norwegian men with a median follow-up of 30 years found that higher baseline SBP levels were associated with an increased risk of atrial fibrillation (16). Individuals with baseline SBP ≥ 140 mmHg or SBP levels of 128–138 mmHg had a 1.6-fold or 1.5-fold risk of atrial fibrillation, respectively, as compared with individuals with SBP <128 mmHg (16). Consistent with the above findings, a secondary analysis of the Ongoing Telmisartan Alone and in Combination With Ramipril Global Endpoint Trial (ONTARGET)/Telmisartan Randomized Assessment Study in ACE Intolerant Subjects With Cardiovascular Disease (TRANSCEND) also reported that the *HR* of AF significantly increased with the levels of baseline SBP, compared with individuals with SBP <120 mmHg (*p* for trend <0.001) (17). Since a higher SBP level was associated with a higher risk of AF, frailty patients with a higher level of SBP were at higher risk of new-onset AF, as compared with those with a lower level of SBP.

The *HR* of the association between frailty and new-onset AF was 1.46 [*95% CI*: (0.81, 2.66)] in patients with prevalent CKD. There are two possible reasons for the insignificance of the correlation. First, the number of new-onset AF in SPRINT was small, and the sample size was insufficient. As a result, although *HR* = 1.46 >1, the correlation was not significant. Second, previous studies found that CKD was an independent risk factor for new-onset AF (18), and patients with CKD were older and had more associated risk factors, which may have led to a reduction in the independent effect of frailty on new-onset atrial fibrillation.

The level of frailty in older adults and the development of AF may share a few common pathological factors, such as strong inflammatory response, low immune function, and neurological damage (19). A previous study found that the cardiac autonomic nervous system (ANS) was impaired in frail older adults, compared with those who are non-frail (20). Sympathetic and vagal activation created the AF substrate, and transient autonomic activation contributed to the dynamic AF substrate, indicating the important role that ANS played in the triggering and maintenance of AF (21). On the one hand, frailty is part of the normal aging process for most people, which may lead to arterial endothelial dysfunction and further result in vascular wall thickening, lumen enlargement, altered tension, stiffness, calcification, inflammation, and reduced regeneration capacity (22, 23), all of which play important roles in the development of AF. On the other hand, normal cardiovascular system aging, such as electrophysiological remodeling and structural changes, may contribute to the development of AF (24).

To our knowledge, this is the first study reporting a positive relationship between the baseline frailty status and new-onset AF. The recognition of frailty status can help cardiologists prevent the development of AF and establish a better prognosis in older adult patients with hypertension. However, several limitations of this study should be discussed. First, the frailty status in this study was obtained from baseline examination with self-examination. A more detailed measurement of frailty changes may provide new insight. Furthermore, the difference in measuring frailty status between this study and the Framingham Heart Study (FHS) Offspring Cohort study restricted us from exploring the reason behind the controversial results between our study and the FHS Offspring study. Further prospective studies were needed to confirm this association and to investigate optimal approaches for preventing the onset of AF in hypertensive older adults with frailty (1).

In summary, baseline frailty status was a strong independent risk factor for new-onset AF among older adult patients with hypertension. Screening for frailty should be considered in older patients with hypertension to prevent new-onset AF. Careful monitoring of older adult patients with hypertension for frailty status may reduce incident AF in this population. Interventions that prevent or delay the progression of frailty, such as muscle strength training and protein supplementation, may also help prevent hypertensive older adults from developing AF.

## Data Availability Statement

The original contributions presented in the study are included in the article/Supplementary Material, further inquiries can be directed to the corresponding author.

## Author Contributions

FH and JC completed the writing of the manuscript. ZW and JY applied for the database and made statistical analysis. YW was responsible for the revision of the manuscript. All authors confirmed the final version of the manuscript.

## Conflict of Interest

The authors declare that the research was conducted in the absence of any commercial or financial relationships that could be construed as a potential conflict of interest.

## Publisher’s Note

All claims expressed in this article are solely those of the authors and do not necessarily represent those of their affiliated organizations, or those of the publisher, the editors and the reviewers. Any product that may be evaluated in this article, or claim that may be made by its manufacturer, is not guaranteed or endorsed by the publisher.

## Footnotes

1
http://www.R-project.org
