# Supplementary material for: Association Between the Frailty and New-Onset Atrial Fibrillation/Flutter Among Elderly Hypertensive Patients
Source: Front Cardiovasc Med. 2022 May 6;9:881946. doi: 10.3389/fcvm.2022.881946 (PMC9120584; doi:10.3389/fcvm.2022.881946)
Supplement: Supplementary file 1 [file Data_Sheet_1.docx]

**Method**

**The evaluation of frailty status according to Fried frailty criteria among SPRINT participants**

The characteristics used in the Fried frailty criteria included weight loss or sarcopenia, slowness, weakness, low activity and self-reported exhaustion. In brief, (1) weight loss or sarcopenia were defined as losing over 10 pounds in the prior year. (2) Slowness was assessed by the 15 feet walking time, and gait speed below or equal to sex- and height-specific 20th percentile was defined as slowness (the height is median in male or female). (3) Weakness was assessed base on grip strength, and defined as below or equal to sex- and BMI-specific 20th percentile. (4) Low activity was assessed by a weighted score of kilocalories expended per week calculated at baseline, based on each participant's report. The lowest quintile of physical activity was identified for each gender. (5) Self-reported exhaustion was identified by two questions from the CES–D scale. A critical mass of characteristics, defined as three or more, had to be present for an individual to be considered frail. Those with no characteristics were considered fit, whereas those with one or two characteristics were hypothesized to be prefrail.

But in the SPRINT population, frailty status cannot be evaluated exactly according to Fried criteria. The main problem was that in the SPRINT, only participants 75 years or older had accomplished a 4-meter walk time, so the frailty status base on Fried criteria was only assessed in those 75 years or older. As for the other four criteria, we can only replace them with similar items in SPRINT. For example, in the SPRINT, patients' weight changes over the past year were not collected at baseline, so we could only use weight loss of more than 10 pounds after the first 12 months of follow-up or low baseline weight (BMI<18.5) to determine the patient as weight loss or sarcopenia. In addition, we use the item "During the past 4 weeks, Have you accomplished less than you would like as a result of your physical health "to evaluate low physical activity. Participants experienced above situation most or all of time were diagnosed as low physical activity. Weakness is evaluated by "Does your health limit you now in climbing several flights of stairs?". Patients’ health limit those daily activity were diagnosed as weakness. Finally, self-reported exhaustion was evaluated with "How much of the time during the past 4 weeks did you have a lot of energy?". Exhaustion was defined as a condition in which patients just sometimes or rarely or never have a lot of energy.

**Table S1. Baseline Characteristics and Crude Outcome According to Frailty Status Assessed By Fried Frailty Criteria.**

| Variables | **Frailty Status** | | | P Value |
| --- | --- | --- | --- | --- |
|  | Fit | Prefrail | Frailty |  |
|  | 0 characteristic | 1-2 characteristics | >=3 characteristics |  |
| Number of participants | 955 | 890 | 128 | - |
| Frailty Index_SPRINT | 0.14 ± 0.06 | 0.20 ± 0.07 | 0.27 ± 0.07 | <0.001 |
| Frailty Status_SPRINT |  |  |  | <0.001 |
| Fit | 225 (23.56%) | 51 (5.73%) | 0 (0.00%) |  |
| Less Fit | 617 (64.61%) | 484 (54.38%) | 23 (17.97%) |  |
| Frailty | 113 (11.83%) | 355 (39.89%) | 105 (82.03%) |  |
| Treatment |  |  |  |  |
| Intensive, n (%) | 456 (47.75%) | 443 (49.78%) | 74 (57.81%) | 0.095 |
| BMI(Kg/m2), mean±SD | 27.37 ± 4.01 | 27.81 ± 4.90 | 30.16 ± 6.75 | <0.001 |
| Age, y |  |  |  |  |
| Overall | 79.05 ± 3.71 | 79.74 ± 4.10 | 80.52 ± 4.08 | <0.001 |
| ≥75y, n (%) | 924 (96.75%) | 869 (97.64%) | 127 (99.22%) | 0.193 |
| Female, n(%) | 330 (34.55%) | 365 (41.01%) | 66 (51.56%) | <0.001 |
| Race, n (%) |  |  |  | 0.003 |
| Non-Hispanic White | 732 (76.65%) | 622 (69.89%) | 87 (67.97%) |  |
| Non-Hispanic Black | 151 (15.81%) | 176 (19.78%) | 33 (25.78%) |  |
| Hispanic | 51 (5.34%) | 76 (8.54%) | 7 (5.47%) |  |
| Other | 21 (2.20%) | 16 (1.80%) | 1 (0.78%) |  |
| Baseline blood pressure, mm Hg |  |  |  |  |
| Systolic, mean±SD | 140.86 ± 15.48 | 142.46 ± 15.85 | 141.62 ± 15.47 | 0.090 |
| Diastolic, mean±SD | 71.73 ± 10.39 | 71.13 ± 10.97 | 70.41 ± 10.99 | 0.279 |
| Heart Rate, bpm, mean±SD | 62.38 ± 10.13 | 64.40 ± 11.33 | 64.05 ± 10.47 | <0.001 |
| Serum creatinine, mg/dL,median (Q1-Q3) | 1.05 (0.89-1.25) | 1.08 (0.87-1.29) | 1.08 (0.91-1.40) | 0.222 |
| Urine Albumin/Creatinine ratio, mg/g Cr, median (Q1-Q3) | 10.87 (6.57-23.45) | 13.21 (7.30-33.58) | 16.48 (9.77-37.24) | <0.001 |
| Estimated GFR, mL min−1 1.73 m−2, median (Q1-Q3) | 64.22 (53.55-76.13) | 63.43 (50.55-75.33) | 58.42 (45.20-72.28) | 0.004 |
| Fasting total cholesterol, mg/dL, mean±SD | 181.45 ± 39.31 | 183.00 ± 36.94 | 183.01 ± 41.12 | 0.672 |
| Fasting total triglycerides, mg/dL, median (Q1-Q3) | 96.00 (70.00-133.00) | 101.00 (73.00-132.00) | 101.00 (72.50-133.25) | 0.343 |
| Fasting HDL cholesterol, mg/dL, median (Q1-Q3) | 53.00 (46.00-65.00) | 53.00 (45.00-63.00) | 53.50 (45.75-66.25) | 0.376 |
| Fasting glucose, mg/dL, mean±SD | 98.26 ± 10.73 | 97.62 ± 12.13 | 98.81 ± 16.31 | 0.376 |
| Statin use, n (%) | 515 (54.21%) | 448 (50.97%) | 63 (49.22%) | 0.288 |
| Aspirin use, n (%) | 596 (62.54%) | 525 (59.06%) | 66 (51.97%) | 0.044 |
| Smoking status, n (%) |  |  |  | 0.008 |
| Never smoked | 492 (51.52%) | 410 (46.07%) | 61 (47.66%) |  |
| Former smoker | 442 (46.28%) | 443 (49.78%) | 61 (47.66%) |  |
| Current smoker | 20 (2.09%) | 37 (4.16%) | 5 (3.91%) |  |
| Previous CVD | 210 (21.99%) | 191 (21.46%) | 32 (25.00%) | 0.664 |
| Previous CKD | 385 (40.31%) | 382 (42.92%) | 67 (52.34%) | 0.031 |
| Framingham 10-y CVD risk score, %, median (Q1-Q3) | 17.38 (11.94-24.19) | 17.67 (11.98-25.46) | 17.14 (11.64-26.62) | 0.161 |
| New-Onset AF, n (%) | 25 (2.62%) | 38 (4.27%) | 7 (5.47%) | 0.076 |

**Table S2. Association between Frailty Status (assessed by Fried frailty criteria) and New-Onset AF in Unadjusted and Adjusted Models.**

|  | **Hazard Ratio (95%CI)** P**-Value** | | |
| --- | --- | --- | --- |
| **Frailty Status** | Model 1 | Model 2 | Model 2 |
| Fit | Ref. | Ref. | Ref. |
| Prefrail | 1.60 (0.96, 2.65) P=0.070 | 1.61 (0.96, 2.70) P=0.071 | 1.50 (0.87, 2.59) P=0.146 |
| Frailty | 2.11 (0.91, 4.90) P=0.082 | 2.21 (0.92, 5.32) P=0.078 | 2.05 (0.83, 5.06) P=0.119 |

Model 1: adjusted for none. Model 2: adjusted for age, sex, race and body mass index. Model 3: adjusted for age, sex, race, body mass index, treatment arms, baseline systolic blood pressure, heart rate, serum creatinine, urine albumin/creatinine ratio, estimated GFR, total cholesterol, triglycerides, HDL-C, glucose, smoking status, statin use, aspirin use, previous CVD, previous CKD , self-reported daibetes, self-reported stroke or TIA, Sokolow-Lyon Index, anemia and Framingham 10-y CVD risk.
